# Cascara Sagrada in Constipation

**Published:** 1879-09

**Authors:** 


### FOR CONSTIPATION.

BUCKTHORN, rhamnus frangula, is a favorite and useful remedy with the profession as a cathartic and laxative, especially in combination with other remedies. From repeated trials we obtain a better effect from rhamnus purshiana, or cascara sagrada, in the form of the fluid extract. An excellent combination, in constipation depending upon an atonic condition of the alimentary canal, is the following:

℞ Fl. Ext. Cascara Sagrada, ℥i.

Ext. Malt.

Syr. Simp. āā ad ℥iv.

M. S. ʒi, ter in die.

